# Bumble bee communities exhibit delayed recovery following severe drought in rangeland

**DOI:** 10.1093/ee/nvag028

**Published:** 2026-04-01

**Authors:** Bethany Roberton, Cameron Duquette, Jason Harmon, Katherine Kral-O’Brien, Torre Hovick, Dillon Fogarty, Travis Seaborn, Kevin Sedivec

**Affiliations:** School of Natural Resource Sciences, North Dakota State University, Fargo, ND, USA; Eastern Oregon Agricultural Research Center, The Nature Conservancy, Burns, OR, USA; Department of Natural Resource Ecology & Management, Oklahoma State University, Stillwater, OK, USA; Department of Natural Resource Ecology and Management, Iowa State University, Ames, IA, USA; School of Natural Resource Sciences, North Dakota State University, Fargo, ND, USA; School of Natural Resource Sciences, North Dakota State University, Fargo, ND, USA; School of Natural Resource Sciences, North Dakota State University, Fargo, ND, USA; School of Natural Resource Sciences, North Dakota State University, Fargo, ND, USA; Central Grasslands Research Extension Center, North Dakota State University, Streeter, ND, USA

**Keywords:** pollinator, grassland, management, climate, resilience

## Abstract

Rangelands offer resources that support bumble bees (*Bombus* spp. [Hymenoptera: Apidae]), which are essential pollinators in agricultural and natural ecosystems. However, relatively little is known on how bumble bee communities respond to and recover from drought within rangeland ecosystems. Our study site in North Dakota experienced an extreme drought in 2021, and we investigated the influences of this drought on bumble bee populations over time. Bumble bee abundance was 98% lower during the drought year compared to the pre-drought year. Abundance increased between 2022 and 2024, but all 3 post-drought years were still lower than the pre-drought year. The average temperatures during the current and previous year’s growing seasons were most strongly associated with abundance patterns. Community composition differed between the pre- and post-drought periods with 3 species identified as pre-drought indicator species. Although we observed increased floral abundance, normal average temperatures, and non-drought PDSI index values in the first-year post-drought, the bumble bee community experienced a lag effect in abundance and species composition recovery. Our research supports the idea that bumble bee communities can be particularly vulnerable to drought, and their recovery can take multiple years, even when conditions quickly return to normal. We found that species with relatively high pre-drought abundance seemed to recover the quickest following the drought, but more research is needed to understand what makes bumble bee species particularly sensitive or resilient. Information like this can help develop management decisions that protect bumble bee communities as droughts become more frequent and severe in the future.

## Introduction

Bumble bees (*Bombus* spp. [Hymenoptera: Apidae]), are important insects that contribute to pollination services in agricultural and natural areas (Aizen et al. 2009, Kleijn et al. 2015, Nayak et al. 2020, Khalifa et al. 2021). These species are extremely productive pollinators with efficient foraging due to their large sizes, variation in tongue lengths, floral constancy, and buzz pollination strategy (Goulson et al. 2008, Kawai and Kudo 2009, De Luca et al. 2013, Pritchard and Vallejo-Marín 2020, Bie et al. 2025). Unfortunately, several factors such as habitat loss and fragmentation, pathogens and parasites, reduced genetic diversity, and pesticide usage are causing worldwide bumble bee declines (Goulson et al. 2008, Cameron et al. 2011, Cameron and Sadd 2020). In the United States, about 54% of bumble bee species have evidence of decline as of 2015, and about 26% are listed as vulnerable, endangered, or critically endangered (Hatfield et al. 2014a, Cameron and Sadd 2020). Bumble bee declines are expected to continue, and climate change has been identified as a major contributor to this loss (Sirois-Delisle and Kerr 2018, Soroye et al. 2020).

Climate change, and particularly drought, can negatively impact bumble bees in a variety of ways which is especially concerning because droughts are expected to increase in frequency and severity in the future (Martinuzzi et al. 2016, Zhao et al. 2020). Drought conditions can directly alter available bumble bee food sources by decreasing floral abundance and species diversity, as well as reducing the number of flowers, floral size, and nectar output of individual plants (Phillips et al. 2018, Rering et al. 2020, Descamps et al. 2021b, Castillioni et al. 2022, Rose-Person et al. 2025). Experiments that simulated drought conditions report that factors such as smaller floral size and decreased number of flowers per plant result in decreased bee visits and decreased floral seed mass and seed set (Rering et al. 2020, Descamps et al. 2021a, Kuppler et al. 2021). Potentially fewer visits to flowers and overall limited resources can cultivate competition between bee species and disrupt plant–pollinator networks (Thomson 2016, Brunet et al. 2025). For example, interspecific competition and loss of available plants may force bees to expend more energy searching for flowers while, at the same time, having a limited subset of floral species to visit (Fontaine et al. 2006, Morozumi et al. 2022). Changes to foraging effort and visitation, alongside altered flowering phenology, further limits visitation between pollinators and available flowers, potentially causing decreased bee diet breadth and nutrition (Memmott et al. 2007, Pyke et al. 2016, Ogilvie et al. 2017).

The influence of climate change on bumble bee community structure as a whole is relatively unknown, but a few major trends have been consistently observed. For one, historical bumble bee surveys compared to present data have tied climate change to altered bumble bee ranges (both expansions and contractions), as well as shifts to higher elevations over time (Cameron et al. 2011, Ploquin et al. 2013, Hindle et al. 2015, Marshall et al. 2020). Another consistently supported trend is that drought contributes to decreases in bumble bee abundance and species diversity (Pyke et al. 2016, Janousek et al. 2023, Scharnhorst et al. 2023). Despite these observed trends in populations, there are specific traits that can determine bumble bee resilience to drought. While many bumble bee species are adapted to cold climates and are therefore vulnerable to heat stress (Soroye et al. 2020, Maebe et al. 2021, White and Dillon 2023), some species are less vulnerable to heat stress due to effective body thermoregulation, water retention, generalist diets, and long foraging flight ranges (Williams 2007, Williams et al. 2009, Maebe et al. 2021). However, resilience to high temperatures can vary even within a bumble bee species (Kenna et al. 2021). It is therefore crucial that research continues to examine impacts of drought conditions on bumble bee communities as a whole and in regards to specific characteristics or features associated with bumble bee species to better understand bumble bee community resiliency and recovery.

Our study adds to current literature about drought impacts on bumble bee and floral communities in rangelands, an underrepresented landscape that is crucial for bee conservation. Rangelands are expansive landscapes dominated by grasses, forbs, or shrubs (Havstad et al. 2007) and provide important floral resources and nesting grounds for bee communities (Krausman et al. 2009, Black et al. 2011, Cane 2011). In return, bumble bee pollination contributes to floral diversity that is essential to rangeland sustainability (Gilgert and Vaughan 2011, Harmon et al. 2011). Rangelands are often used for livestock grazing (Fleischner 1994, Rinehart 2006), so most research regarding bees in rangelands examines the impact of grazing (Shapira et al. 2020, Cutter et al. 2021, Goosey et al. 2024). However, the use of rangelands for supporting bee communities has limited research in the Northern Great Plains, and few studies are able to incorporate spontaneous drought events into analysis because of the unpredictability of climate conditions. Here, we leverage ongoing research on bumble bee communities in North Dakota rangelands to examine bumble bee responses to an extreme drought in 2021. Across 5 yr of data collection (pre-drought 2018, drought 2021, and post-drought 2022 to 2024), we targeted 2 objectives:

1. Determine bumble bee abundance before, during, and after drought, and assess the relative importance of floral abundance and growing season weather conditions for explaining observed abundance patterns.
2. Quantify bumble bee community compositional responses to drought and identify bumble bee characteristics or patterns associated with resilience to drought.

## Methods

### Study Site

This study took place at North Dakota State University’s Central Grassland Research Extension Center (CGREC) located near Streeter, North Dakota in Kidder County (46°45′N, 99°28′W). The CGREC is located in the Prairie Pothole Region and has main soil types Zahl-Williams loams, Zahl-Max-Bowbells loam, and Zahl-Williams-Zahill complex (Soil Survey Staff WSS 2025). This site is in the major land resource area 53B with ecological sites within the claypan, clayey, sandy, and loamy groups (Sedivec et al. 2021). The site is characterized as a mixed-grass prairie and is dominated by western wheatgrass (*Pascopyrum smithii* [Rydb.] Á. Löve), green needlegrass (*Nassella viridula* [Trin.] Barkworth), and blue grama (*Bouteloua gracilis* [Willd. ex Kunth] Lag. ex Griffiths) (Patton et al. 2007, Limb et al. 2018). It also has high cover of the non-native grasses Kentucky bluegrass (*Poa pratensis* L.) and smooth brome (*Bromus inermis* L.) (Patton et al. 2007). The most abundant woody plant is western snowberry (*Symphoricarpos occidentalis* Hook.) (Limb et al. 2018). The forb community is diverse and includes many species such as milkweeds (*Asclepias* spp.), goldenrods (*Solidago* spp.), coneflowers (*Echinacea* spp.), sages (*Artemisia* spp.), thistles (*Cirsium* spp.), yellow sweet clover (*Melilotus officinalis* L.), alfalfa (*Medicago sativa* L.), prairie rose (*Rosa arkansana* Porter), and yarrow (*Achillea millefolium* L) (Rogers et al. 2005, Limb et al. 2018, Duquette et al. 2022). Additional forbs often found at the site are purple prairieclover (*Dalea purpurea* Vent.), hairy goldaster (*Heterotheca villosa* [Pursh] Shinners), leadplant (*Amorpha canescens* Pursh), wild licorice (*Glycyrrhiza lepidota* [Nutt.] Pursh), cinquefoils (*Potentilla* spp.), and sunflowers (*Helianthus* spp.) (see Sedivec et al. 2021 for more reference plant communities within the site's resource area). The average temperature for CGREC ranges from −12.5 °C to 22.0 °C with a mean annual precipitation of 504 mm, ranging from 11 to 31 mm per month from November through April, and 70 to 92 mm per month from May through October (NDAWN 2025).

We conducted surveys in 8, 65-ha pastures managed with cattle grazing. The original intent of this ongoing research was to examine the impact of grazing strategy on bumble bee communities. Although we do not compare between strategies in this paper, we included a description of the treatments here for clarity. Half of the pastures were grazed under a patch-burn grazing (PBG) framework, while the other half were managed with season-long grazing (SLG). These pastures are further designated into 8 subplots of 8-ha each. The PBG treatment is burned on a 4-yr rotation with a quarter of each pasture being burned every spring (Duquette et al. 2022). SLG reflects conventional grazing management and has cattle graze continuously from late May through late October with full pasture access (Sanderson et al. 2015). The stocking rates each year for both treatments were based on achieving 40% to 50% utilization.

### Drought Conditions in 2021

In 2021, CGREC was part of widespread drought conditions as quantified by the US Drought Monitor. The US Drought Monitor is based out of the University of Nebraska-Lincoln (UNL) and is produced through a partnership between the National Drought Mitigation Center at UNL, the US Department of Agriculture, and The National Oceanic and Atmospheric Administration that provides maps, data, and classifications of drought conditions across the United States. The drought classifications are as follows: D0 (abnormally dry), D1 (moderately dry), D2 (severe drought), D3 (extreme drought), and D4 (exceptional drought). On a weekly evaluation from May through August of 2021, 47% to 85% of North Dakota land area experienced extreme drought (category D3) and 8% to 17% of the state experienced exceptional drought (category D4). Our study site’s county experienced a range in 70% to 100% of land area at extreme drought during this same timeframe and 19% of land area at exceptional drought during the month of August in 2021.

**Fig. 1.**
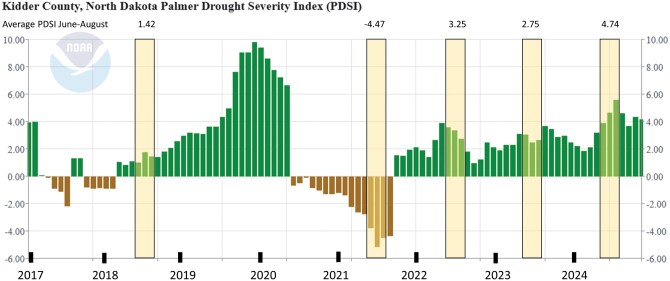
The PDSI values for Kidder County, North Dakota, from January 2017 through December 2024 obtained from NOAA. Our survey months (June to August) are highlighted in yellow for each survey year with the average PDSI values above during those months. Figure and data accessed from NOAA on 25 July 2025. Moisture categories: extremely wet (4.00 and above), very wet (3.00 to 3.99), moderately wet (2.00 to 2.99), slightly wet (1.00 to 1.99), near normal (0.49 to −0.49), mild drought (−1.00 to −1.99), moderate drought (−2.00 to −2.99), severe drought (−3.00 to −3.99), and extreme drought (−4.00 and below) (Palmer 1965, Heim 2002).

The monthly Palmer Drought Severity Index (PDSI) values for Kidder County from 2017-2024 (NOAA 2025, Fig. 1) additionally quantifies these drought conditions. PDSI measures the duration and intensity of long-term drought to create moisture categories: extremely wet (4.00 and above), very wet (3.00 to 3.99), moderately wet (2.00 to 2.99), slightly wet (1.00 to 1.99), near normal (0.49 to −0.49), mild drought (−1.00 to −1.99), moderate drought (−2.00 to −2.99), severe drought (−3.00 to −3.99), and extreme drought (−4.00 and below) (Palmer 1965, Heim 2002). During our survey months for each year at our study sites (June to August), 2021 was the only year below a zero index with an average index of −4.47, an extreme drought (Fig. 1).

### Bumble Bee Surveys

We integrated 2 bumble bee datasets collected at the CGREC from 2018 and 2021 to 2024 to examine bumble bee response to drought across multiple years. All bumble bees from the datasets were identified from specimens or photographs by C. Duquette and B. Roberton using taxonomic keys (Williams et al. 2014, Arduser 2020, Ascher et al. 2025). We collected both datasets across the same pastures. Every year, we conducted surveys on each 8-ha (20 acre) subplot in PBG (32 subplots) and SLG (32 subplots). All 64 subplots were surveyed across 3 rounds in June, July, and August of each year for a total of 192 surveys every year except for 2024. In 2024, we surveyed 36 out of 64 subplots in the third round giving 164 total surveys that year due to unfavorable weather conditions and unavailable surveyors. The 36 subplots surveyed were chosen at random and were evenly split between the 2 grazing management strategies. We had shared survey methods across years including utilizing the same subplots, catching bumble bees that are found on flowers (excludes fly-throughs or nesting bees), and identifying the associated flower species. However, there were some methodological variations in floral survey locations and search effort. We therefore have separated the survey method details below by years to explain these variations in detail. We then explain how we accounted for these differences during analysis in the data analyses section.

### 2018 Surveys (Pre-Drought Reference Year)

Each 8-ha subplot had 2, 50-m by 50-m plots that were surveyed for 20 min for a total search time of 40 min per subplot in 2018. Within each plot survey, 10 min were spent conducting a systematic grid search in 3 transects and 10 min were spent examining flowers for bumble bees with a bias toward areas with dense floral resources. A bee was caught if it was on a flower, and the clock was stopped whenever a bee was caught to be processed. Each bee was collected to be identified later.

### 2021 to 2022 Surveys (Drought Year and First-Year Post-Drought)

We established one, 100-m transect placed roughly in the middle of each subplot in 2021 and 2022. We surveyed each transect searching for bumble bees within 1 m on each side. In total, this contributed to approximately 10 min of search time within each subplot. Bumble bees that were caught visiting flowers were photographed for identification and released live to reduce lethal collections (Montero-Castaño et al. 2022).

### 2023 to 2024 Surveys (Second- and Third-Year Post-Drought)

Bee surveys were modified in 2023 and 2024 to increase search effort due to detecting low bumble bee abundance during the drought year and the first post-drought year. We followed the same 100-m transects that were used in 2021 and 2022 with the same transect survey methodology. However, following each transect survey, a 50-m by 50-m plot was searched alongside the transect line for 10 min, with a bias toward areas with dense floral resources. The clock was stopped whenever a bee on a flower was caught to be processed. In total, this contributed to approximately 20 min of search time within each subplot (about 10 min of search time from the transect survey followed by 10 min of search time from the plot survey). Bumble bees that were caught were photographed for identification and released live.

#### Floral Survey Methods

Floral surveys were conducted each year to quantify what flowering plants were available for bumble bees to visit during the growing season. In 2018, floral surveys were conducted once a week in each 8-ha subplot along a 300-m transect. All individual flowering plants were counted within 2.5 m on each side of the transect. These surveys were done once a week from May through August giving 15 survey rounds. Following every transect survey from 2021 to 2024, we conducted a floral survey following the same 100-m transect used for bumble bee surveys in each 8-ha subplot. All individual flowering plants were counted within 2.5 m on each side of the transect. These surveys were conducted in 3 rounds each summer for a total of 192 surveys each year except for the third round in 2024 (36 completed out of 64 for this final round to give 164 total surveys for this year).

### Data Analysis

All analyses were performed in R 4.4.2 (R Core Team 2024). Figures were created using the function *ggplot* in the ggplot2 package (Wickham 2016). Results were considered statistically significant if *P* ≤ 0.05.

#### Objective 1

We used generalized linear mixed models (GLMM) to examine bumble bee abundance between years using the glmmTMB package (Brooks et al. 2017) and checked residuals with the DHARMa package (Hartig 2024). We examined both Poisson and negative binomial families for the count data and used negative binomial because it had a better fit that accounted for overdispersion (dispersion= 0.36, *P*-value = 0.84). In our preliminary analyses, we examined bee abundance across transect surveys alone for 2021 to 2024, removing abundance from the plot surveys conducted in 2023 and 2024 to determine if added plot surveys skewed our abundance results for those years. We thought there may be potential for differences in survey methods to skew the data because plot surveys allow for more flexibility in following and catching a bumble bee, and therefore more bees may be caught in plot rather than transect surveys. However, our data does not show that adding plot surveys with free search time in 2023 and 2024 significantly skewed the results because the comparison of bee abundance across only the transect surveys from 2021 to 2024 was similar to abundances when bees from plot surveys were added.

For abundance across years, we used counts of bumble bees (both transect and plot surveys included) as the response variable, year as the fixed effect, and round and pasture as random effects to account for repeated measures (round) and replicate (pasture). Our model included an offset argument with a log of search time in minutes to account for differences in search effort between years (Appel et al. 2021, Raderschall et al. 2022, Milberg et al. 2025). We used the *emmeans* function in the emmeans package (Lenth 2025) to perform posthoc pairwise comparisons following statistically significant results from the GLMM. We additionally ran this model with subplot as a random effect and had similar results (similar *P*-values from the GLMM results and same significant differences between years following post hoc analyses), so we removed the subplot effect to keep a simpler model.

Flowering plant abundance was examined across years using a GLMM. Because longer transects and more floral surveys were conducted in 2018, we kept 12 out of the 15 rounds of surveys that were conducted between June and August during 2018. We divided the 2018 floral counts by 4 (to account for 4 surveys done each month in comparison to one survey done each month during 2021 to 2024) and then by 3 (to account for the 300-m transect in comparison to the 100-m transects done in 2021 to 2024). We used floral counts as the response variable, year as the fixed effect, and pasture and round as the random effects. We used the tweedie family distribution to account for continuous data skewed to the right, and we checked for overdispersion with the DHARMa package (dispersion = 0.35). We used the *emmeans* function in the emmeans package (Lenth 2025) to perform post hoc pairwise comparisons following statistically significant results from the GLMM.

We assessed the relative importance of selected weather variables on bumble bee abundance using a model selection approach to understand impacts of drought. All variables used were from the survey months (June, July, and August) of each survey year and the previous year. We included the PDSI from Kidder County, ND, for each month (NOAA 2025), the average monthly temperature (°C), average daily rainfall (mm) for each month, and total monthly rainfall (mm) (NDAWN 2024, Streeter Station). We ran a correlation test across all weather variables using the *cor* function and removed correlated variables (>0.6). The average daily rainfall values for each month (both current survey year and previous year) were correlated with the total monthly rainfall (both current survey year and previous year), so we removed the average daily rainfall values altogether. We kept the total monthly rainfall variable because we thought this factor would have more detectable differences since most daily averages of rainfall were close to zero.

Our original GLMM examining bee abundance across years from above was used as the null. To create candidate models, we used all non-correlated variables as fixed effects along with the fixed variable (year), offset (log for search time), and random effects (pasture and round) from the null. We tested combinations of models that best fit our hypotheses of temperature, rainfall, and PDSI of the current survey year and previous year explaining abundance patterns (see Table 1 for all tested variable combinations). Model support was compared using Akaike Information Criteria corrected for small samples sizes (AICc) with model assessments based on ΔAICc values (values 0 to 2 indicate strong relative support) (Bolker 2008). We used the *AICctab* function in the bbmle package (Bolker et al. 2023) to run all models and calculate AICc values and weights. We kept the top models (ΔAICc < 2) and selected their coefficients as the explanatory factors for our abundance results. The variables identified as explanatory coefficients were then graphed and analyzed with an ANOVA using the *aov* function to compare between years and to normal (30-yr averages) weather variables obtained from the Streeter NDAWN Station as applicable.

**Table 1.** All models ran with explanatory weather variables and their corresponding AICc, ΔAICc, and model weight

Top models	AICc	ΔAICc	Df	Weight
**Survey year average temperature**	**490.5202**	**0**	**9**	**0.311436**
**Previous year average temperature**	**491.5411**	**1.02095**	**9**	**0.186927**
**Survey year and previous year average temperature**	**492.0982**	**1.578045**	**10**	**0.141482**
**Previous year average temperature, total rainfall, and average PDSI**	**492.2314**	**1.711179**	**11**	**0.132371**
**Survey year average temperature, total rainfall, and average PDSI**	493.4588	2.93861	11	0.071657
**Null**	494.1721	3.6519	8	0.050161
**Survey year average PDSI**	494.7802	4.259985	9	0.037011
**Survey year total rainfall**	496.3995	5.879353	9	0.01647
**Previous year total rainfall**	496.4956	5.975439	9	0.015697
**Previous year average PDSI**	496.5016	5.981386	9	0.01565
**Survey year and previous year average PDSI**	496.912	6.391801	10	0.012747
**Survey year and previous year total rainfall**	498.721	8.200766	10	0.005159
**Survey year average temperature, total rainfall, and average PDSI** **AND** **Previous year average temperature, total rainfall, and average PDSI**	499.6569	9.136723	14	0.003231

The top 4 (ΔAICc < 2) are in bold. Weather variables were assessed as monthly values: average temperature (°C), PDSI, and total rainfall (mm) from June to August of each survey year and previous year.

#### Objective 2

We examined compositional differences in bee communities among years with a PERMANOVA within the *adonis2* function in the vegan package (Oksanan et al. 2025) after we confirmed that dispersion was homogenous between factor groupings with the *betadisper* function (*P* = 0.755). For this objective, we removed 27 total bees that did not have a confirmed identification because they were observed on a flower but flew away before being caught and identified (removed 2 in 2021, 1 in 2022, 6 in 2023, and 18 in 2024). We also removed any species that had only 1 individual observed in total across all survey years (for our study, this was only *Bombus pensylvanicus* De Geer [Hymenoptera: Apidae]). Following the PERMANOVA, we used the *pairwise.adonis* function in the pairwiseAdonis package (Martinez 2017) to look at comparisons between years, and we visualized the data with an NMDS using the *metaMDS* function in the vegan package (Oksanen et al. 2025). For the NMDS, we combined the species abundance for 2021 and 2022 due to the low number of bumble bees caught during those 2 yr. We visually examined the community ordination with an NMDS (*k* = 2, stress = 0.15) and created 2 groups to show the “before drought” period (2018) versus the “after drought” period (2021 to 2024).

Next, we ran an indicator species analysis with the function *multipatt* in the indicspecies package (De Cáceres 2009) to see if any bumble bee species were associated with specific survey years. Indicator species analyses analyze the relationship between a species abundance from a set of sampled sites to select species indicative of a specific given category, such as treatment or year (De Cáceres 2025). In short, it selects species that reflect the current state of their environment and provide evidence of the impacts of environmental change (De Cáceres 2025). We chose this analysis because we specifically wanted to see if there are bumble bee species associated with our surveys before, during, or after the drought as an indicator of diversity changes during this time period.

Finally, we created a table of our detected bumble bee species with their IUCN Red List status, selected characteristics, yearly abundance, and adjusted abundance to account for search time (2018 abundance divided by 4, and 2023 and 2024 divided by 2 to give counts per 10 min of search time). We utilized the IUCN Red List (Hatfield et al. 2014a, b, c, d, 2015a, b, c, d, e, f, g, h), the Bumble Bee Watch (a community science project created by Xerces Society and partners [2025]), an identification guide on Bumble Bees of North America (Williams et al. 2014), and a statewide survey of bumble bees in North Dakota (Pei et al. 2022) to create a condensed characteristics table. We chose characteristics that are sensitive to environmental pressures (Williams et al. 2009, Lozier et al. 2021, Fitzgerald et al. 2022) and that we therefore believe may influence whether a bumble bee species recolonizes after drought. Table 2 lists our chosen characteristics with our hypotheses on which specific traits may be more or less resilient to drought conditions (Svensson et al. 2000, Goulson et al. 2005, 2006, 2008, Williams et al. 2009, Bartomeus et al. 2013, Wood et al. 2019, Grass et al. 2021, Liang et al. 2021, Martinet et al. 2021, Gérard et al. 2022). These include associated habitat types, location of North Dakota within a species’ known range (edge vs. more central), peak month of activity for different guilds, queen body size, and tongue length. The queen body categories we are using are small (length range mostly below 18 mm), medium (length range falls within 18 to 22 mm), and long (length range mostly above 21 mm). We determined North Dakota's location within a species’ range based on species distributions maps developed by the IUCN (Hatfield et al. 2014a). To clarify tongue length, there is not a specific length range of the bee tongue, or glossa, that defines these categories. The short, medium, or long categories are instead based on the lengths of the 4 segments of the labial palps in relation to each other. For example, in long-tongued bees, the first 2 segments of the labial palps are much longer than the last 2 (Wilson and Messinger Carril 2016).

**Table 2.** The selected characteristics that may be sensitive to environmental change, determine resiliency to drought, and indicate the ability to recolonize our site following a severe drought event

Characteristic	Hypothesized resiliency and higher chance of recolonization after drought		Supporting details
	More resilient	Less resilient	
**Associated habitat types**	Species that mostly or solely use grassland habitat	Species not associated with grassland habitat	Bumble bees tend to not be habitat specialists (Goulson et al. 2006) but species’ queens show some preference in nest-site selection (Svensson et al. 2000)
**North Dakota Location Within Range**	North Dakota is more central in known range	North Dakota is on edge of known range	Species found on the edges of climatic ranges versus the middle are more susceptible to decline (Williams et al. 2009).Species at edge of geographic range more sensitive to environmental change (Goulson et al. 2005)
**Peak activity month (queen, worker, and male)**	Species with early (May) active queens, early worker foraging, and early reproduction	Species with late (June) active queens, late worker foraging, and late reproduction	Late-emerging species with queens that become active later in the year are more susceptible for decline due to loss of food plants for mid to late colony development and lack of nest sites taken by early-emerging species (Goulson et al. 2005, 2008, Williams et al. 2009).
**Queen body Size (mm)**	Small and medium body sizes	Large body size	Larger body sizes can be more sensitive to higher temperatures and depend on more abundant and nutritious floral resources (Bartomeus et al. 2013, Grass et al. 2021, Martinet et al. 2021, Gérard et al. 2022)
**Tongue length (short, medium, long)**	Short and medium tongues	Long	Longer tongued bees tend to have more specialized diets and are more susceptible to decline while short to medium tongued bees have more generalist diets and seem more resilient (Goulson et al. 2005, Wood et al. 2019, Liang et al. 2021)

We included our hypotheses for which specific characteristics will be more resilient or less resilient to drought with the supporting details used to create these hypotheses from scientific literature.

## Results

### Objective 1

**Fig. 2.**
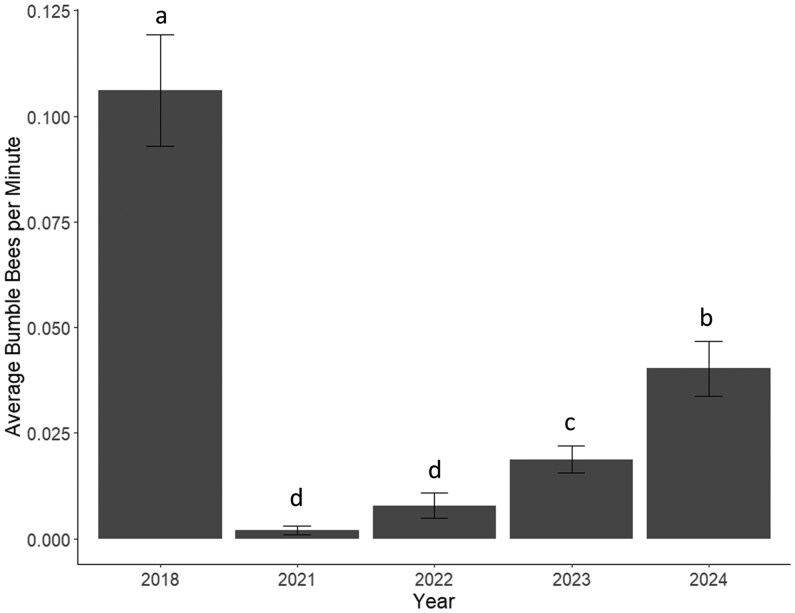
The average number of bumble bees caught per minute during each of the 5 survey years (2018, 2021 to 2024) with standard error. We calculated bees/minute to visually represent the offset of search time that is included in our model. The letters indicate significance between years (*P* < 0.05).

**Fig. 3.**
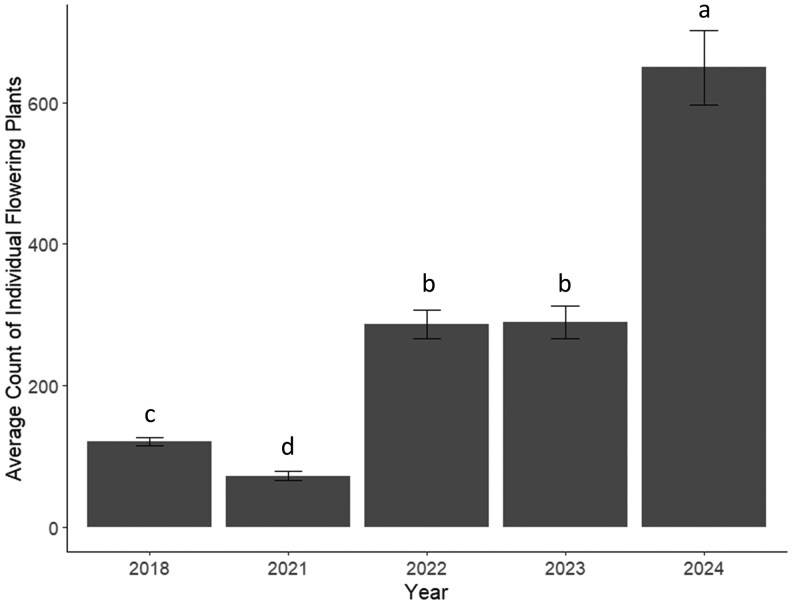
The average count of individual flowering plants from floral surveys for each year (2018, 2021 to 2024) with standard error. To account for differences in survey methods, 2018 floral counts were divided by 4 (4 surveys done per month in 2018 vs. 1 survey done per month in the other years) and then divided by 3 (300-m surveys done in 2018 vs. 100-m surveys in other years). To average floral counts, we divided the total counts from the entire summer of each year by the number of conducted surveys (2018, 2021, 2022, and 2023 were divided by 192, and 2024 counts were divided by 164).

We detected a total of 1,038 bumble bees visiting flowers (815 in 2018, 4 in 2021, 15 in 2022, 72 in 2023, and 132 in 2024). After accounting for search time, abundance was roughly 204 individual bees in 2018, 4 in 2021, 15 in 2022, 36 in 2023, and 66 in 2024. Abundance exhibited a 98% decrease in 2021 compared to 2018. From 2021 to 2024, abundance increased by a factor of 16.5, but was still only 32% of pre-drought abundance in 2018. We graphed average bumble bees per minute to account for search time and visually represent the reported abundances (Fig. 2). We found flowering abundance to be significantly lowest during the drought year in 2021, followed by the pre-drought year 2018 (Fig. 3). Flowering abundance increased significantly the first year after the drought in 2022 and increased again in 2024 (Fig. 3).

**Fig. 4.**
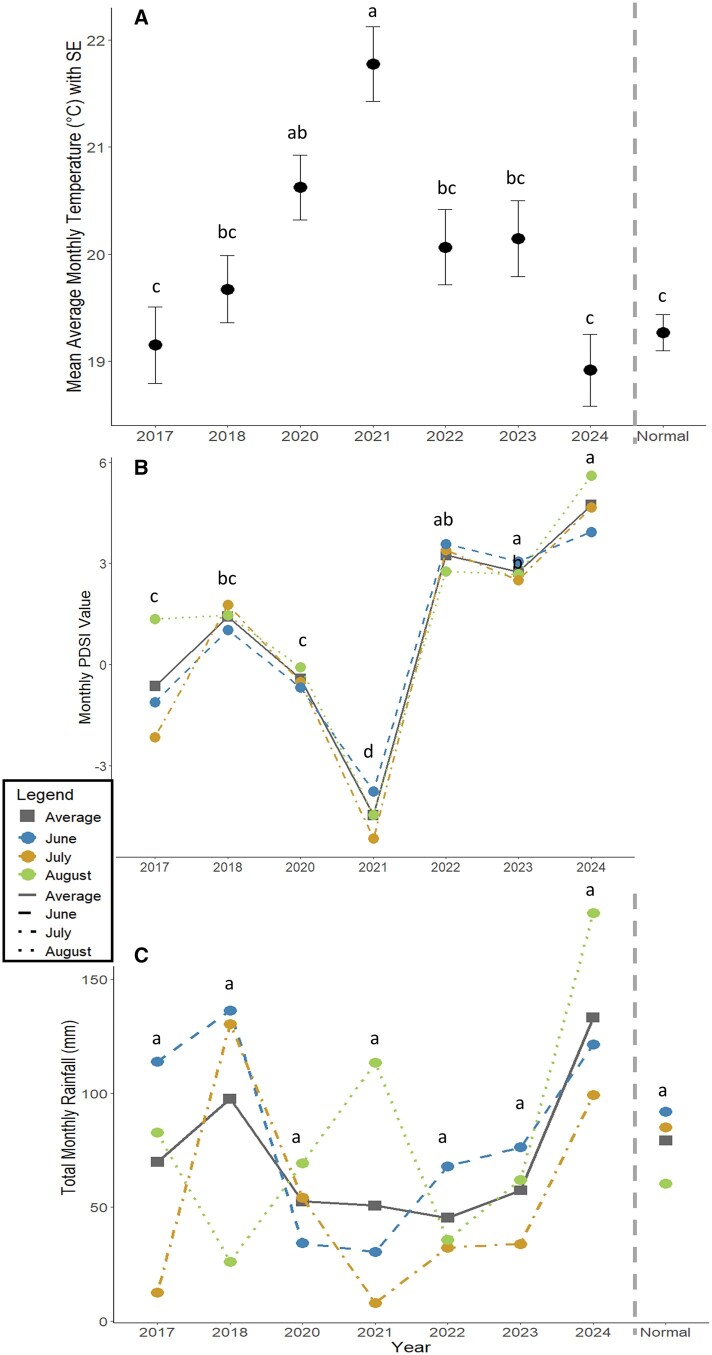
Weather variables obtained for June-August for each survey year (2018, 2021, 2022, 2023, and 2024) and the previous year: (A) mean average monthly temperature (°C) with standard error (NDAWN 2025), (B) monthly PDSI values (NOAA 2025) and (C) total rainfall per month (mm). The normal variables shown for temperature and rainfall are 30-yr averages for the Streeter site (NDAWN 2025). The gray lines with squares in the PDSI and rainfall graphs are the average across the 3 mo for each year. The letters indicate significance (*P* < 0.05).

We identified 4 top models (Table 2), with the first 2 having the strongest influence after examining their confidence intervals (not overlapping zero). The average temperature of the survey year explained the most variation and had a negative influence on bumble bee abundance (β = −0.31, SE = 0.12, 95% CI = −0.55 to −0.06). The next most explanatory variable was the average temperature of the previous year, which had a weak positive influence (β  =  0.19, SE = 0.08, 95% CI = 0.02 to 0.36). The temperatures from June to August for 2021 were higher (*P* ≤ 0.05) than all other years (except for 2020 which was not significantly different from 2021) as well as the long-term average for our site (Fig. 4A). PDSI did not have strong explanatory power for either the current survey year (β = −0.37, SE = 0.28, 95% CI = −0.93 to 0.19) or the previous year (β  =  0.01, SE = 0.11, 95% CI = −0.22 to 0.24), although the average PDSI from June to August was lowest (*P* ≤ 0.05) in 2021 compared to all other years (Fig. 4B). Average total rainfall for survey year (β = −0.0008, SE = 0.002, 95% CI = −0.006 to 0.004) and the previous year (β = −0.0006, SE = 0.005, 95% CI = −0.01 to 0.01) also did not have strong explanatory power. Average total rainfall across June to August was not significantly different between any years or from the normal values (Fig. 4C).

### Objective 2

**Fig. 5.**
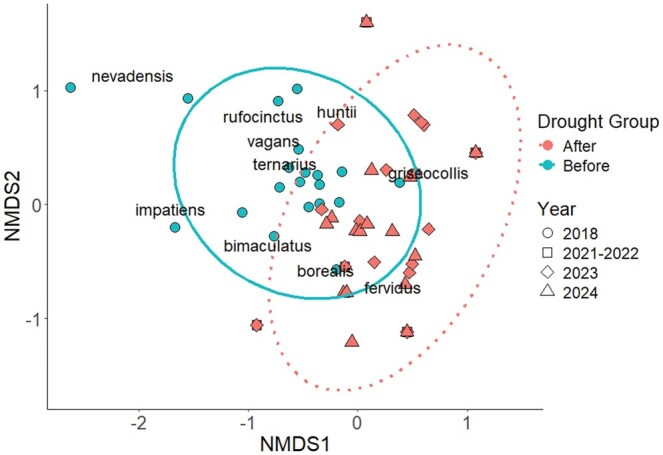
Non-dimensional metric scaling (NMDS) of bumble bee community composition between significant groups: before drought and after drought (PERMANOVA *P* = 0.001). Before drought is the year 2018 while after drought encompasses 2021 to 2024. Bumble bee species were extracted from NMDS data scores and added to visually show species driving group differences. Each year is represented by a shape: 2018 = circle, 2021 to 2022 = square, 2023 = diamond, and 2024 = triangle.

We detected a total of 11 species, with ten species found in 2018, 2 in 2021, 4 in 2022, 6 in 2023, and 7 in 2024 (Table 3). Out of the 10 bumble bee species detected before the drought in 2018, 7 have been detected again between 2021 and 2024. The 3 species that have not been detected again post-drought were *Bombus nevadensis* Cresson (Hymenoptera: Apidae), *B. rufocinctus* Cresson, and *B. impatiens* Cresson. Additionally, one species listed as vulnerable was newly detected in 2024, *B. pensylvanicus* (which was not included in the PERMANOVA analysis or NMDS visualization due to having only one individual across all 5 yr of surveys). We found a difference (*P* ≤ 0.05) in the bumble bee community composition due to survey year (PERMANOVA *P* = 0.001, *F* = 4.5973), with pairwise comparisons indicating that the before drought community (2018) was different (*P* ≤ 0.05) from the community during drought and post-drought years. We visually showed the community ordination of the before and after communities (Fig. 5).

**Table 3.** Bumble bee species detected from 2018 and 2021 to 2024 surveys with their IUCN Red List status and habitat types (Hatfield et al. 2014a, b, c, d, 2015a, b, c, d, e, f, g, h), main characteristics (Williams et al. 2014, Pei et al. 2022, Bumble Bee Watch 2025) and yearly abundance

Bumble bee species	IUCN red list status	Main habitat types	Where North Dakota falls in range	Month of peak activity			Queen body size range (mm) and size rank (small, medium, large)	Tongue length Category	Abundance (Adjusted counts in parentheses to account for varying search times)				
				Queen	Worker	Male			2018	2021	2022	2023	2024
B. huntii** **Greene**	LC	SL, GL, AT	Eastern edge	6	7.5	9	19–20 Medium	Medium	304 (76)	0	4	11 (6)	11 (6)
B. borealis** **Kirby**	LC	FR, SL, GL, WL, AT	Middle to southern edge	6	6.5	8	18–22 Medium	Long	223 (56)	1	2	10 (5)	35 (18)
B. griseocollis* **De Geer**	LC	GL, WL, AT	Northern edge	6.5	7	7.5	18–23 Medium	Short	159 (40)	1	5	30 (15)	42 (21)
B. fervidus*** **Fabricius**	VU	FR, SL, GL, AT	Middle	5.5	8	8.5	18–21 Medium	Long	53 (13)	0	3	11 (6)	22 (11)
B. ternarius* **Say**	LC	FR, WL	Middle to southern edge	6	8	9	17–19 Medium	Medium	40 (10)	0	0	3 (2)	2 (1)
B. bimaculatus* **Cresson**	LC	FR, SL, WL, AT	Northwestern edge	5.5	7	7.5	18–22 Medium	Medium	13 (3)	0	0	0	1 (1)
B. nevadensis** **Cresson**	LC	GL, AT	Eastern edge	5.5	7	7.5	24–25 Large	Long	9 (2)	0	0	0	0
B. rufocinctus** **Cresson**	LC	FR, SL, GL, AT	Middle	6	7.5	9	17–18 Small	Short	7 (2)	0	0	0	0
B. impatiens* **Cresson**	LC	FR, SL, WL, AT	Very western edge, typically only detected in southeastern corner	5.5	7.5	10	21–23 Large	Medium	4 (1)	0	0	0	0
B. vagans** **Smith**	LC	FR, WL, AT	Middle	6	8	8.5	17–21 Medium	Medium	3 (1)	0	0	1 (1)	0
B. pensylvanicus*** **De Geer**	VU	GL, AT	Northern edge	6	8.5	9	22–26 Large	Long	0	0	0	0	1 (1)

The IUCN Red List categorizes species into least concern (LC), near threatened (NT), vulnerable (VU), endangered (EN), critically endangered (CR), extinct in the wild (EW), and extinct (EX). The species categorized as least concern with relatively stable populations in the United States are indicated with one asterisk*, the species categorized as least concern with evidence of declining populations in the United States are indicated with 2 asterisks** and the species that are categorized as vulnerable with evidence of declining populations in the United States are indicated with 3 asterisks***. The habitat types identified in the IUCN Red List assessments include forest (FR), shrubland (SL), grassland (GL), wetland (WL), and artificial/terrestrial (AT). Additional characteristics chosen were where North Dakota falls within that species range, the month of peak activity for the queens, workers, and males, queen body size range (mm), and tongue length category (short, medium, and long). The yearly abundances from the surveys are given in raw counts but have adjusted counts for search time in parentheses to compare abundance per 10 min. Species are in order of most abundant to least abundant during the pre-drought year 2018.

The indicator species analysis found 3 species, *B. borealis* Kirby, *B. ternarius* Say, and *B. huntii* Greene, associated with the pre-drought community (2018). All 3 of these species were relatively abundant before the drought with a total of 142 individuals after accounting for search time, making up 70% of all bumble bees caught that year. All 3 were also detected again in at least 2 of the post-drought years with a total of 45 individuals after accounting for search time from 2021 to 2024, making up 41% of all bumble bees during this timeframe. No bumble bee species were uniquely associated with any other year. Besides having relatively high abundances in the pre-drought year, 3 characteristics appear to be associated with resilience or being detected again following drought: medium queen body size, later peak queen activity (June), and a relatively short window between queen and worker peak activity month (1.5 months or less) (Table 3).

## Discussion

We conducted 5 yr of bumble bee surveys to quantify bumble bee abundance and community composition before, during, and after a severe drought in North Dakota rangeland. We found that drought negatively impacted bumble bee abundance and potentially contributed to alterations in community composition when compared to a reference pre-drought survey year. Most notably, we report that bumble bee communities need multiple years to recover from a drought event in terms of abundance and number of species. The species that seemed to recover the quickest and were detected again at our site had the highest abundances in the pre-drought year and 3 characteristics associated with queens. Our results support that drought conditions, and in particular, growing season temperature, is associated with negative impacts on bumble bee communities, but there is potential to identify resilient species that can recover in the years following drought.

Our study is not the first to note decreased native bee abundance associated with drought or with hotter and drier conditions in general (Iserbyt and Rasmont 2012, Soroye et al. 2020, Neria et al. 2025). We expected abundance to decrease during the drought year because drought has been previously associated with fewer forager observations and lower nest density (Gallagher and Campbell 2017, Thuma et al. 2025). However, our data demonstrate that bumble bees exhibit a lagged recovery following severe drought which is not commonly observed due to the few opportunities to study communities during and after a drought. We demonstrate this pattern using 3 key findings: (i) growing season average temperature for the current and previous year having associations with our observed bumble bee abundance patterns, (ii) return of near normal temperatures the year following the drought, and (iii) rapid recovery of floral abundance following drought.

The best supported weather models identified average temperature of the survey months not only for the current year but also for the year prior to explain our bumble bee abundance patterns. Similarly, another study found that ground-nesting bees (including bumble bees) had delayed emergence associated with weather conditions of the current and previous year (Neria et al. 2025). Specifically, we observed that the survey year growing season average temperatures had a negative influence on abundance while previous year growing season average temperature had a slight positive influence. This may be because bumble bee abundance began to increase 2 yr following the drought as average temperatures began to return closer to normal after the drought. As for our other tested variables, the average PDSI was significantly lowest in 2021 compared to all other years indicating that this variable was associated with the abrupt decline in bumble bee abundance but was a poor predictor of abundance patterns across non-drought years. In other words, drought indices like PDSI may have weak or negligible effects on bumble bee abundance until a threshold is crossed (ie an extreme drought) and abrupt losses occur. Despite drought conditions, total rainfall did not differ among years and was a poor predictor of bumble bee abundance patterns. Precipitation has been found to be weakly related to occupancy of bumble bee species in North America, but occupancy is strongly related to temperature (Jackson et al. 2022). This is not to say that lack of precipitation during a drought is not an important influencing factor. The month of July during the 2021 drought had an average daily rainfall of 0.25 mm and total monthly rainfall of 8 mm, while August of 2021 had an average daily rainfall of 3.6 mm and total monthly rainfall of 113.5 mm (which was higher than the month of August for both 2018 and 2022). Thus, similar to PDSI, bumble bee abundance is likely sensitive to seasonal anomalies in rainfall but largely unresponsive across normal ranges. Additionally, we only examined weather conditions for the growing season to address this objective, but weather variables from the dormant season may have also impacted bumble bee abundance patterns or floral community growth.

Although temperature was associated with abundance, we did not see an immediate increase in bumble bee abundance when temperatures returned back to near normal in the first-year post-drought. The average temperature stayed near normal for each of the 3 yr following the drought, but bumble bee abundances lagged even after 3 yr post-drought. This result indicates other factors potentially influence bumble bee community recovery. Native bee communities in general are sensitive to changes in floral resources (Roulston and Goodell 2011), so it is possible that drought impacts on the floral community are contributing to the observed lag effect. However, we found that floral abundance increased in the first year following the drought, surpassing floral abundance for even the pre-drought year of 2018. Therefore, future research should examine floral diversity and species composition across survey years in conjunction with abundance. It is important to also note that bumble bees may be susceptible to lag effects after a major drought event due to their life history traits. Nests are founded by a single queen with mostly monandrous reproduction and are thus susceptible to low effective population sizes (Chapman and Bourke 2001, Goulson et al. 2008, Cameron et al. 2011, Owen and Whidden 2013). Bumble bees rely on adequate nutrition for queens to produce nests, and colonies continue to need nutritious floral resources during their entire season to contribute to reproductive success for the next year (Carvell et al. 2015, Goulson et al. 2018, Vaudo et al. 2018, Hollis Woodard et al. 2019). Bumble bee dependence on a growing season’s resources to create a population in the following year would help explain the lag effect we observed as well as the composition of the bumble bees following the drought.

Bumble bee community composition differed between the pre-drought year (2018) and all other years together (2021 to 2024). A study in California also reported differences in native bee composition between pre- and post-drought periods, showing a negative impact of drought on native bee assemblages (Hung et al. 2021). In total, we detected 11 bumble bee species with 10 of those being found in the pre-drought year. Two of the 11 total species are listed as vulnerable according to the IUCN Red List (*B. pensylvanicus* and *B. fervidus* Fabricius*)*, and 7 identified as declining or having evidence of some decline in the United States *(B. pensylvanicus, B. fervidus, B. vagans* Smith, *B. huntii, B. borealis, B. nevadensis*, and *B. rufocinctus* Cresson*)* (Evans et al. 2008, Jepsen 2008, Cameron et al. 2011, Colla et al. 2012, Hatfield et al. 2014a, b, c, d, 2015a, b, c, d, e, f, g, h). During the drought year, we detected 2 species, *Bombus borealis* and *Bombus griseocollis.* Both of these species are common, larger-bodied bees with generalist diets and little evidence of declines; they were also relatively abundant in our pre-drought survey year. These life history traits may have therefore contributed to their ability to withstand the drought at sufficient numbers to be detected or to at least continue to utilize the flowers that remained available at the site.

We identified 3 bumble bee species associated with pre-drought conditions from the indicator species analysis (*B. borealis, B. ternarius*, and *B. huntii*). No other species were found to be associated with the drought year or the 3 yr following this analysis, further supporting a shift in community composition that lingered after drought. However, there were 3species that were only observed in 2018 (*B. nevadensis, B. impatiens*, and *B. rufocinctus*). Very few individuals of these species were caught in 2018 which may be why they were not identified by the indicator species analysis. Because of the low numbers associated with our pre-drought year, we expect the drought made it especially difficult for these species to recover at our site. Altogether, we considered 5 species to be most common at our site and potentially exhibit higher resiliency to drought conditions. These species had at least 10 individuals detected in 2018 and had more than one individual detected again after 2021. This list includes *B. griseocollis*, a common and generalist diet bee species, *B. fervidus*, a listed vulnerable and declining species (Hatfield et al. 2015d), and the 3 species that were associated with 2018 (*B. borealis, B. ternarius*, and *B. huntii)*. To better understand why these 5 species were the most abundant of all our 11 detected species, we compared species abundance patterns and characteristics to see if any seemed to be indicative of a species being found again after severe drought.

Our results highlight 3 characteristics shared among most of the 5 species that recovered and therefore may be indicators of a species’ ability to recolonize an area after drought (besides all 5 having relatively high abundances prior to drought): (i) medium queen body sizes (all 5 species), (ii) peak queen activity in June (4 out of the 5 species), and (iii) a short period of time between queen peak active period and worker peak active period (3 species had 1.5 months or less between these 2 periods). Of the 6 species that were less abundant in our study, 3 had early queen peak activity (May). The window between queen and worker peak activity months were longer for all 6 of these species (1.5 months or longer). All other characteristics including main habitat type, North Dakota’s location within a species’ range, male peak activity month, and tongue length did not appear to have any obvious patterns between our most common and less common species.

We believe that pre-drought abundance may be one of the strongest determining factors in resilience to drought conditions at our site. This is because bumble bees in general tend to have low effective population sizes, making lower populations more susceptible to extirpation events (Goulson et al. 2008), and there were no species with low pre-drought abundances that had more than one individual detected again after the drought. Another study examining bumble bee nest density in drought years found that the common species at their site recovered quickly in the first year after drought (Thuma et al. 2025), so species abundance before major drought may be important for recovery afterwards. As far as queen body size, over half of all eleven detected species were of medium size, so this characteristic may not be a reliable trait when examined by itself for determining resiliency. The peak activity patterns are interesting because they did not necessarily fit our original hypotheses. We hypothesized that the earliest active bumble bees would be more resilient to drought because of their potential to create nesting sites and use early resources (Goulson et al. 2005, 2008, Williams et al. 2009), but the more resilient species we identified typically had later active queens in June rather than May. This may be a result of North Dakota’s climate with warmer temperatures and more abundant floral species in June than in May. On a related note, a study in Britain found that grassland areas favored later-emerging queens due to flowering occurring later (May to July) (Edwards and Williams 2004). The additional short window between queen and worker active periods may be a beneficial trait as well where workers are most active quickly following nest establishment. Although all these characteristics stand out for the more resilient species we identified, the pattern of high pre-drought abundance seems to be the most consistent especially as the 3 characteristics are also found amongst the other species that did not seem to be as resilient in some way or another. For example, there were 3 of the less resilient species that also had June active queens, but all 3 of these species are documented as declining (Cameron et al. 2011, Colla et al. 2012, Hatfield et al. 2014a), which may have contributed to their inability to recolonize. It may therefore be combinations of these characteristics that together favor certain species. Other studies have also found difficulty in distinguishing overarching characteristics that explain observed community patterns; a combination of factors is likely contributing to species-specific responses to drought (Goulson et al. 2005, Williams et al. 2009). However, continuous evaluation of species-specific traits can help determine bee responses to varying kinds of disturbance and influence management decisions (Williams and Osborne 2009).

Our study compared bee and floral data across 5 yr and included a 2-yr gap without survey data between 2018 and the 2021 drought. Despite this gap in survey data, we feel our results accurately characterize bumble bee abundance before, during, and after drought. Our site has been utilizing the same land management strategies since before our first survey year of 2018, which provides a relatively consistent land management legacy for the time period of our study. Therefore, any major differences in bumble bee communities (or floral communities) detected between years are more than likely not a result of management practices. Moreover, we have data from the drought year as well as the 3 yr following, giving us a reliable timeframe to examine trends in bumble bee abundance and composition. Although we compare these trends to a single pre-drought year, we used 2018 as a reference rather than baseline for “normal” bumble bee communities at our site. We emphasize that the slow recovery of the bumble bee community following the drought is our main focus and is the most important finding.

Moving forward, it would be valuable to continue bumble bee surveys at our site to better understand abundance and community composition patterns for additional years following drought. Two bumble bee species listed as vulnerable have been detected at the site (Hatfield et al. 2014a), including *B. pensylvanicus*, which has North Dakota at the very northern edge of its range with only one individual detected in 2024 (third-year post drought). Three of the species on our list (*B. borealis, B. vagans*, and *B. ternarius*) have been specifically identified as being negatively impacted by temperature change in the past 120 yr and are more cool-adapted species (Jackson et al. 2022). As temperatures continue warming, southern species may shift to more northern states, and we may detect more individuals of southern associated species in the future. In general, continued monitoring of bumble bee community responses to drought conditions is needed to better understand how communities will be impacted by a changing climate. Higher temperatures not only decrease bumble bee abundance but can alter foraging behavior and survival rates (Guiraud et al. 2021, Gérard et al. 2022). Although cold-climate adapted bumble bee species tend to be more impacted by warming temperatures (Martinet et al. 2021, Miller-Struttmann et al. 2022, Hemberger and Williams 2024), warm-adapted species also suffer local declines and changes in range distribution (Kerr et al. 2015, Hemberger et al. 2021).

Our research supports that bumble bee communities are negatively impacted by drought and may require several years following a severe drought to recover in terms of abundance and community composition. As the severity and frequency of drought are expected to increase in in the future (Cook et al. 2018, Vicente-Serrano et al. 2022), concerns for bumble bee communities should focus on vulnerable species and the apparent lag effect after drought. Since we did not find single defining characteristics of resilient bumble bees to drought, it may be more difficult to predict how each species will respond. However, abundance prior to drought appears to be an important factor for bumble bee communities to recover after drought and focusing on supporting bumble bee abundances may be key to facilitating resiliency to drought events. Rangelands will be essential in supporting species as they respond to drought since they provide diverse nesting and floral resources over large areas, and thus, potentially more space for foraging and resource partitioning (Westphal et al. 2006, Cane et al. 2011, Gilgert and Vaughan 2011). These landscapes also allow for lower exposure risks to pesticide usage and close proximity to additional habitats in adjacent natural areas and croplands (Black et al. 2011, Cane et al. 2011, Gilgert and Vaughan 2011). Current research on rangeland management strategies suggests that utilizing grazing and/or prescribed fire can promote resource diversity to support bee communities (Vulliamy et al. 2006, Lázaro et al. 2016, Shapira et al. 2020, Keyser et al. 2022, Goosey et al. 2024). We therefore plan to next explore the impacts of the 2 grazing strategies utilized at our research site on the bumble bee community during and after the 2021 drought. As we further monitor bumble bees in these landscapes, it will be increasingly important to understand how these areas can support the resilience of pollinator communities to drought.

## Data Availability

Data and code used in this manuscript are available through Dryad: https://doi.org/10.5061/dryad.bk3j9kdrx
